# Tricuspid repair in mitral regurgitation surgery: a systematic review and meta-analysis

**DOI:** 10.1186/s13019-023-02158-9

**Published:** 2023-02-17

**Authors:** João Lopes Cardoso, Gonçalo Nuno Ferraz Costa, Fátima Neves, Lino Gonçalves, Rogério Teixeira

**Affiliations:** 1 Serviço de Cardiotorácica, Centro Hospitalar Vila Nova de Gaia/Espinho, Rua Conceição Fernandes, 4434-502 Vila Nova de Gaia, Portugal; 2 Serviço de Cardiologia, Centro Hospitalar E Universitário de Coimbra, Coimbra, Portugal; 3 Faculdade de Medicina da Universidade de Coimbra, Coimbra, Portugal; 4 Coimbra Institute for Clinical and Biomedical Research (iCBR), Coimbra, Portugal

**Keywords:** Tricuspid valve, Mitral valve, Tricuspid regurgitation, Prophylactic tricuspid repair

## Abstract

**Background:**

Concomitant tricuspid repair in MR surgery is indicated in patients with severa tricuspid regurgitation, however, concomitant repair in less-than-severe TR patients is still a matter of debate.

**Methods:**

In December 2021, we systematically searched PubMed, Embase and Cochrane databases for randomised control trials (RCTs) comparing isolated MR surgery versus MR surgery with concomitant TR annuloplasty. Four studies were included, resulting in 651 patients (323 in the prophylactic tricuspid intervention group and 328 in the no tricuspid intervention group).

**Results:**

Our meta-analysis showed a similar all-cause mortality and perioperative mortality for concomitant prophylactic tricuspid repair when compared with no tricuspid intervention (pooled odds ratio (OR), 0.54; 95% confidence interval (CI): 0.25–1.15, *P* = 0.11; I^2^ = 0% and pooled OR, 0.54; 95% CI: 0.25–1.15, *P* = 0.11; I^2^ = 0%, respectively) in patients undergoing MV surgery. despite a significantly lower TR progression (pooled OR, 0.06; 95% CI: 0.02–0.24, *P* < 0.01; I^2^ = 0%). Additionally, similar New York Heart Association (NYHA) classes III and IV were identified in both concomitant prophylactic tricuspid repair and no tricuspid intervention, despite a lower trend in the tricuspid intervention group (pooled OR, 0.63; 95% CI: 0.38–1.06, *P* = 0.08; I^2^ = 0%).

**Conclusions:**

Our pooled analyses suggested that TV repair at the time of MV surgery in patients with moderate or less-than-moderate TR did not impact on perioperative or postoperative all-cause mortality, despite reducing TR severity and TR progression following the intervention.

**Supplementary Information:**

The online version contains supplementary material available at 10.1186/s13019-023-02158-9.

## Background

Tricuspid regurgitation (TR) is a common condition and is observed in 0.55% of the general population: its prevalence increases with age and affects 4% of individuals over 75 years old [1]. A secondary aetiology represents the majority of cases and is associated with left-sided valvular or myocardial dysfunction. Secondary TR may also develop later after left-sided valve surgery [1]. Additionally, late reoperation for severe TR in patients with right heart failure is associated with high perioperative mortality [2, 3]. Therefore, a growing consensus suggests that severe TR should be addressed during index procedures, particularly in symptomatic patients [4]. These recommendations are largely based on observational data [5, 6].

The operative approach for less-than severe TR is widely debated. Mild or moderate TR, not corrected at the time of left-sided cardiac surgery, may progress in 25% of patients leading to poorer late survival and functional outcomes [5, 7]. Risk factors for TR progression include annular dilation measuring 40 mm or more in diameter on preoperative transthoracic echocardiography, right ventricular (RV) dysfunction and the presence of leaflet tethering, pulmonary hypertension, atrial fibrillation or transvalvular pacing or defibrillator leads [7–9].

Accordingly, wide variations exist for managing less-than-severe TR at the time of left-sided cardiac surgery: the frequency of tricuspid-valve (TV) repair at the time of mitral-valve surgery ranges from 5 to 75% [10].

In our systematic review and meta-analysis, we assessed the prognostic benefits and interventional risks associated with TV repair at the time of MV surgery in patients with moderate or less-than-moderate TR.

## Methods

### Study protocol and registration

This study was designed according to the Preferred Reporting Items for Systematic Reviews and Meta-Analyses (PRISMA) statement (Additional file 1: Table S2). The study was also registered in the International Prospective Register of Systematic Reviews database (CRD42022296613).

### Study selection criteria/eligibility criteria

Only studies investigating adult populations (≥ 18 years old) with moderate TR or less than moderate TR with annulus dilation were included. Studies with secondary mitral regurgitation (MR), primary TV disease and endocarditis were excluded. We focused on trials that used placement of tricuspid annulus ring or suture-type annuloplasty (e.g., DeVega annuloplasty) techniques at the time of MV intervention. Partial annuloplasty techniques were excluded (e.g., Kay annuloplasty). We included randomised controlled trials (RCTs) with parallel-group designs and excluded single-arm studies. We also excluded all cohort and case–control studies, and the following: reviews, dissertations, theses, editorials, study protocol, clinical guidelines, commentaries and letters. Only studies that used medical therapies as comparators were included. Studies had to assess individual outcomes of all-cause mortality, perioperative mortality (30-day mortality post-surgery), reoperation for TR, TR progression by two grades and the presence of moderate-severe TR or patients with a New York Heart Association (NYHA) class III–IV condition.

### Information sources and search strategy

The search strategy was devised by GC and JC. Bibliographic databases were systematically searched. RCTs on tricuspid repair in MV surgeries were extracted and merged with hits from the bibliographic database search. Electronic database searches were complemented by searching clinicaltrials.gov to capture results from ongoing or recently completed RCTs not yet published. We also scanned the reference lists of eligible studies to capture additional trial articles by cross-referencing. The search strategy is shown (Additional file 1: Table S2). We conducted searches in PubMed, Embase and Cochrane Central Register of Controlled Trials databases.

The search strategy included the following terms and all variants in multiple combinations in each database: "mitral valve repair," "mitral valve surgery," and "tricuspid insufficiency." Standard search terms for RCTs were also used wherever possible. No planned search restrictions in the search strategy were used to prevent overlooking important studies that were not correctly classified in the respective bibliographic databases. Databases were searched from database inception, without time restrictions. We also limited our searches to English language studies. Literature searches were updated during peer-review to include the most up to date literature.

### Data collection and management

Study selection and data extraction were performed in duplicate by two reviewers. Both were blinded to each other’s decisions but not to journal titles, study authors, or institutions. Mendeley software was used to store, organise and manage references, and allow a transparent and reproducible systematic search. Both reviewers independently scanned study titles and abstracts against eligibility criteria. Full texts were acquired from studies meeting inclusion criteria. Reviewers’ results were compared and when disagreements occurred, critical points were discussed until a consensus was reached. If necessary, other study authors were asked to resolve eligibility issues. Reasons for excluding trials were fully documented. After eligible studies were selected, two reviewers independently extracted predefined data from the abstracts and full texts. Extracted items included first author, publication year, country, number of participants, sex, mean/median age, race/ethnicity, mean/median body mass index, echocardiographic mitral and tricuspid parameters, type of procedure and cardiovascular risk factors. If summary data were not published, they were calculated from study data.

### Quality assessments

All planned statistical analyses were registered in PROSPERO before data collection to preclude data-driven analyses and the selective reporting of statistically significant findings. The study protocol was developed in line with the Preferred Reporting Items for Systematic Review and Meta-Analysis Protocols (PRISMA-P), the Cochrane Handbook for Systematic Reviews of Interventions. We adhered to current PRISMA guidelines.

### Descriptive and meta-analyses

Outcomes were addressed by estimating odds ratios (ORs) and 95% confidence intervals (CIs). Results of the intention-to treat (ITT) approach were used, including all patients randomised when both ITT and per-protocol results were provided. Effect estimates from eligible studies was assessed with random-effects model through the DerSimonian and Laird method (primary analysis). Heterogeneity was visually represented using forest plots and statistically assessed by Cochran’s Q test (significance level = 0.05) and the I^2^ index (< 25% low, 25–50% moderate and > 50% high heterogeneity). A meta-analysis was conducted even if high heterogeneity was detected, with results discussed within a heterogeneous context. Heterogeneous sources were explored using sensitivity analyses; we excluded studies with a high or unknown risk of bias according to the Cochrane RoB-2 assessment tool for randomised trials. The quality assessment for each study is presented in the “risk of bias summary” (Table 1) Publication bias was visually represented in funnel plots and tested using the Egger’s test (Additional file 1: Figures S1–S4). The certainty of the evidence was evaluated using the GRADE approach (Additional file 1: Table S3).

**Table 1:**
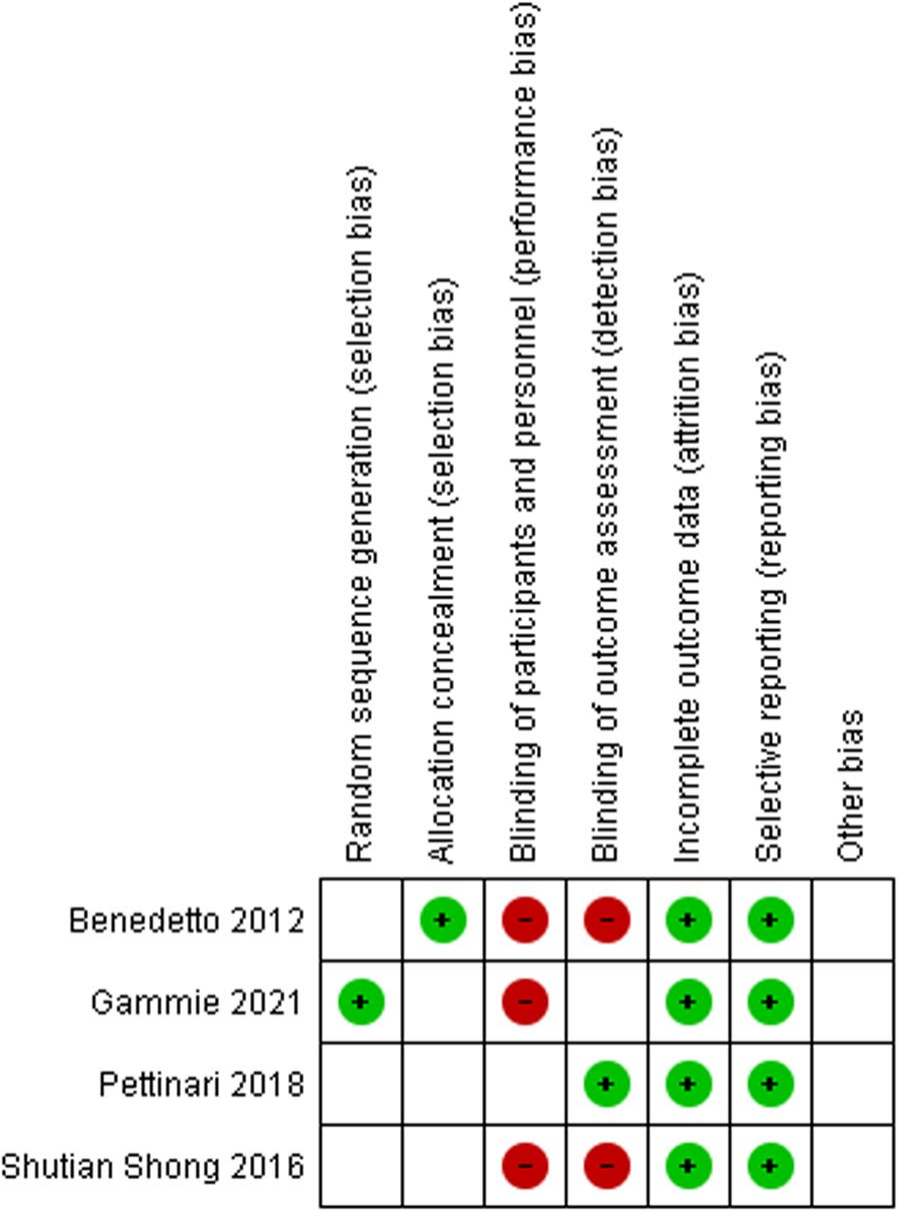
Risk of Bias Summary

“ + ” means low risk of bias; “-“ means high risk of bias

## Results

### Search results

**Fig. 1:**
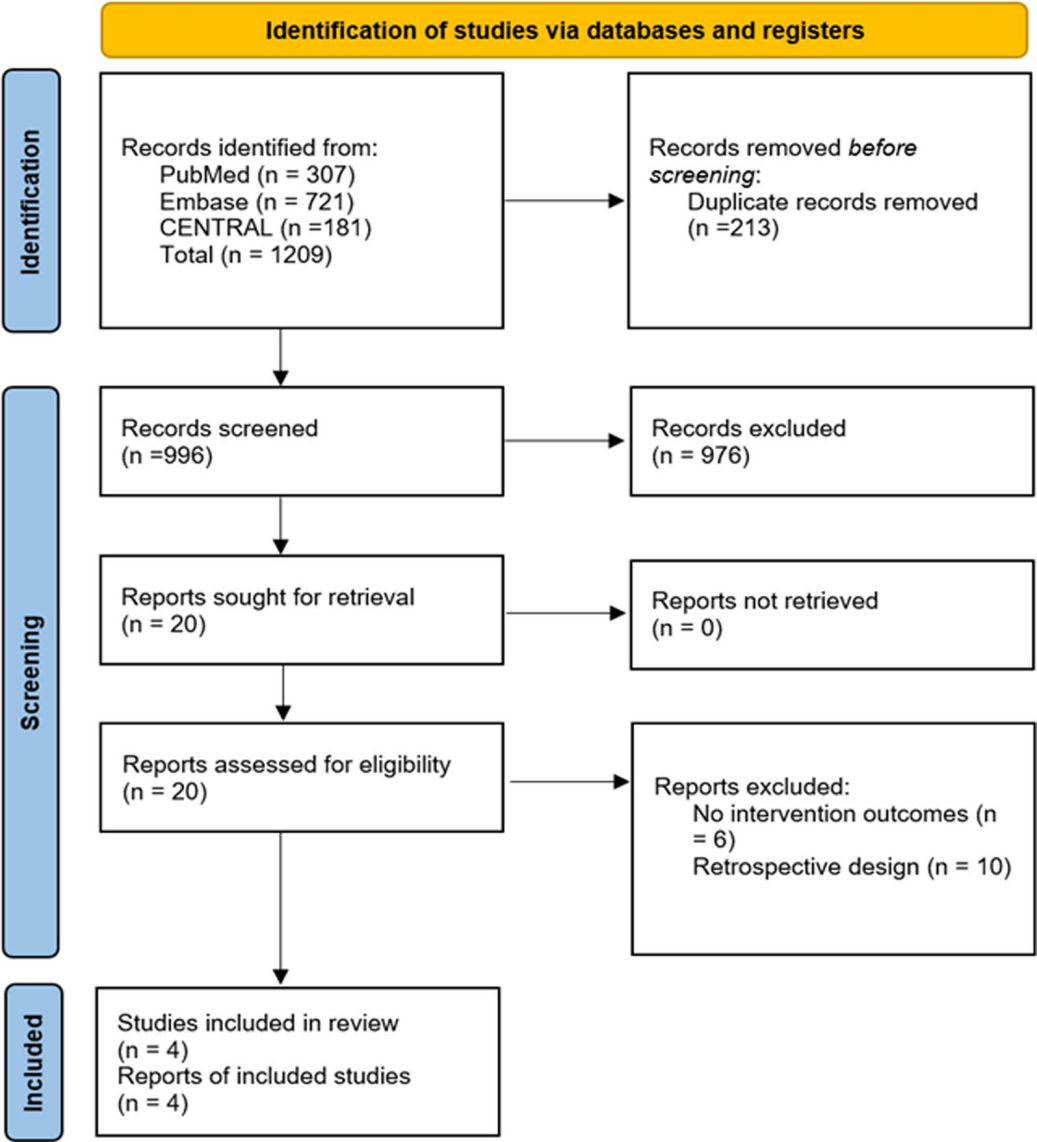
Literature search flow diagram

We identified 1209 articles from our literature search. After duplicate removal, we excluded 996 publications based on titles and abstracts, study type and study population assessments (Fig. 1). Four studies were finally included, resulting in 651 patients (323 in the prophylactic tricuspid intervention group and 328 in the no tricuspid intervention group). Study characteristics are described (Table 2) and patient baseline characteristics are outlined (Table 3).

**Table 2:** Study Characteristics

Study	Year of Publication	Design	Follow-up Time	Tricuspid Regurgitation Criteria	Annular Dilation Criteria	Tricuspid Valve Repair Technique	Number of patients	
							Tricuspid Intervention	No Tricuspid Intervention
Gammie et al	2021	Randomised trial	2 years	Moderate or < Moderate TR with Annular dilatation of 40 mm or more (or 21 mm/m^2^)	Yes	Technique at surgeon’s discretion with use of a rigid, incomplete, nonplanar ring	198	203
Pettinari et al	2018	Randomised trial	3.8 years	< Severe functional TR (< 7 mm VC)	No	Carpentier approach with semi-rigid annuloplasty ring	53	53
Song et al	2016	Randomised trial	2 years	Mild TR (Area of regurgitation ≤ 4 cm ^2^)	No	De Vega surgery or Carpentier ring fixation	50	50
Benedetto et al	2012	Randomised trial	1 year	< Moderate (< + 2) functional TR and dilated tricuspid annulus (≥ 40 mm)	Yes	Flexible Cosgrove-Edwards annuloplasty system	22	22

*TR* Tricuspid Regurgitation

**Table 3:** Demographics and Baseline Characteristics of Included Patients;

Author	Number of patients		Mean age		Female gender (%)		LVEF (%)		Degenerative Cause (%)		Tricuspid Dimension (mm)		Cardiopulmonary bypass time (min)		Atrial Fibrillation preoperatively (%)		Concomitant Procedures	
	TI	No TI	TI	No TI	TI	No TI	TI	No TI	No TI	TI	TI	TI	TI	No TI	TI	No TI	TI	No TI
Gammie et al	198	203	66.6 ± 10.7	68.2 ± 9.7	24.6%	25.8%	64.1 ± 7.1	64.3 ± 7.4	100%	100%	42 ± 4.6	42.2 ± 4.7	166.1 ± 69.3	132.6 ± 58.8	43.9	44.3	53.7	53
Pettinari et al	53	53	64.2 ± 13.7	62.1 ± 14.5	32.1%	37.7%	58 ± 9	59 ± 10	58.5%	62.3%	40.1 ± 5.8	39.6 ± 5.4	123 ± 35	100 ± 36	32	21	43	47
Song et al	50	50	46.6 ± 5.7	47.8 ± 6.5	54%	50%	NR	NR	NR	NR	NR	NR	105.1 ± 25	99.5 ± 26.7	NR	NR	NR	NR
Benedetto et al	22	22	64 ± 15	68 ± 19	40%	50%	51 ± 8	55 ± 7	36%	36%	43 ± 3	44 ± 3	139 ± 37	120 ± 66	40	54	50	73

*LVEF* Left Ventricular Ejection Fraction, *TI* Tricuspid Intervention, *NR* Not Reported

### Study outcomes

**Fig. 2:**
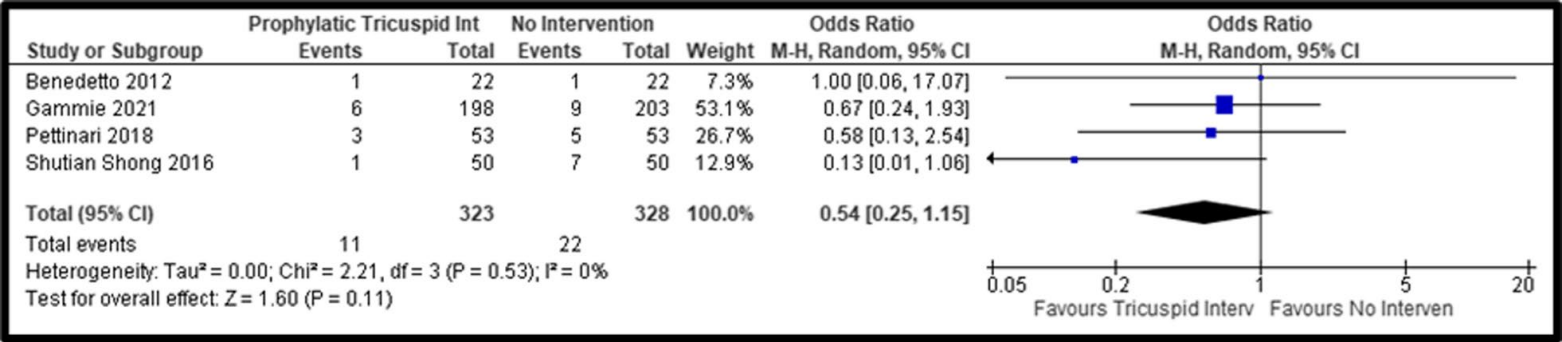
Forest Plot of All-Cause Mortality comparing Tricuspid Repair versus No Intervention; *CI* Confidence Interval; *M–H* Mantel–Haenszel

**Fig. 3:**
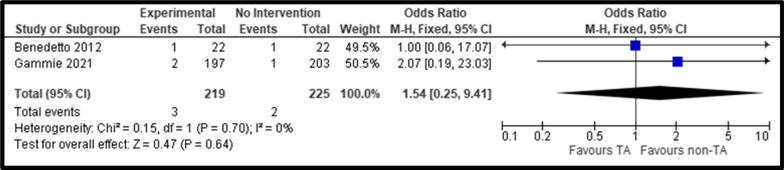
Forest Plot of Perioperative Mortality comparing Tricuspid Repair versus No Intervention; *CI* Confidence Interval; *M–H* Mantel–Haenszel

Overall, we identified a similar postoperative all-cause mortality for concomitant prophylactic tricuspid repair when compared with no tricuspid intervention (pooled OR, 0.54; 95% CI: 0.25–1.15, *P* = 0.11; I^2^ = 0%) (Fig. 2). In terms of perioperative mortality, no significant differences were observed (pooled OR, 0.54; 95% CI: 0.25–1.15, *P* = 0.11; I^2^ = 0%) (Fig. 3).

**Fig. 4:**
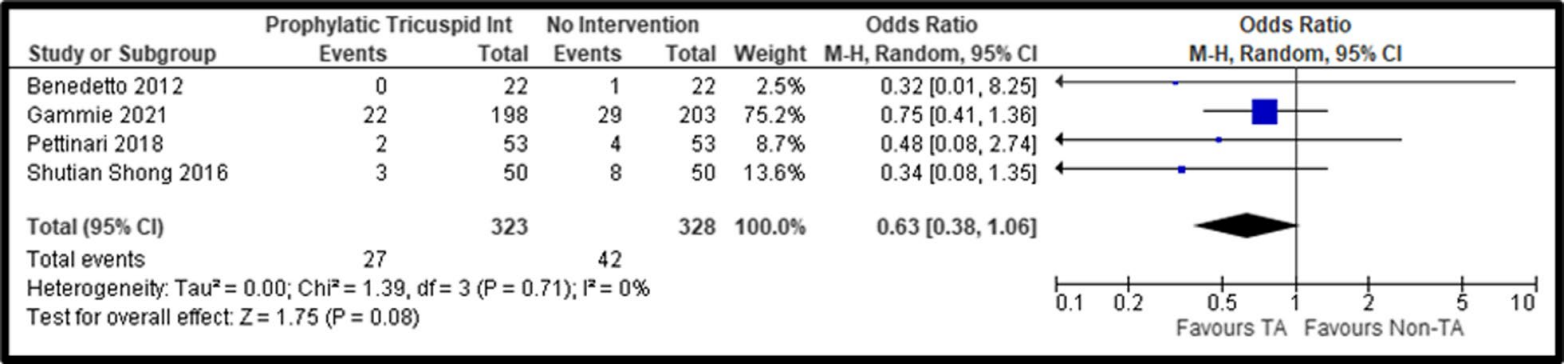
Forest Plot of NYHA III–IV comparing Tricuspid Repair versus No Intervention; *CI* Confidence Interval; *M–H* Mantel–Haenszel; *NYHA* New York Heart Association

**Fig. 5:**
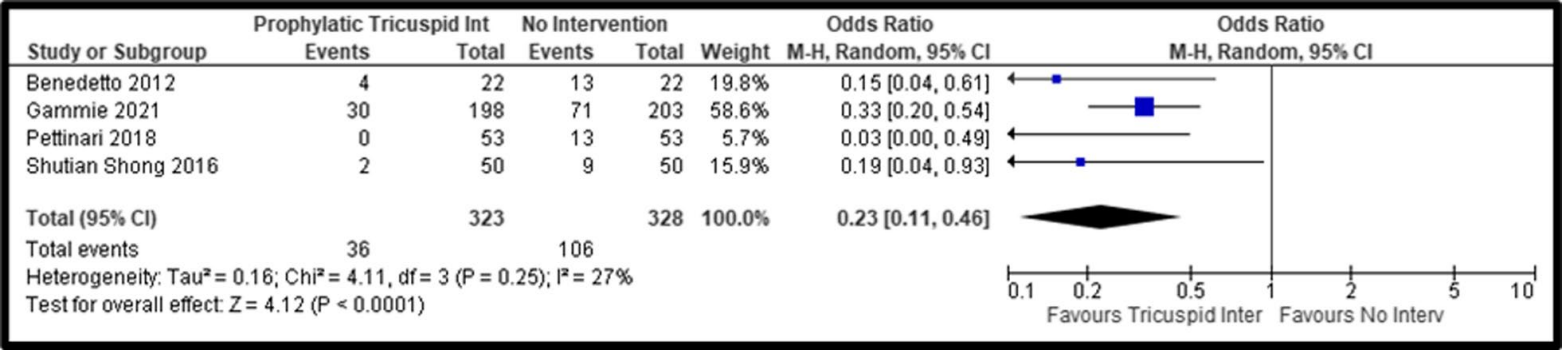
Forest Plot of TR moderate-severe condition at follow-up comparing Tricuspid Repair versus No Intervention; *CI* Confidence Interval; *M–H* Mantel–Haenszel; *TR* Tricuspid Regurgitation

Additionally, we identified similar NYHA III–IV classes in both groups, despite a lower trend in the tricuspid intervention group (pooled OR, 0.63; 95% CI: 0.38–1.06, *P* = 0.08; I^2^ = 0%) (Fig. 4). We identified a significantly lower TR progression (pooled OR, 0.06; 95% CI: 0.02–0.24, *P* < 0.01; I^2^ = 0%) and postoperative moderate-severe TR (pooled OR, 0.23; 95% CI: 0.11–0.46, *P* < 0.01; I^2^ = 27%) (Fig. 5) in the group that underwent prophylactic tricuspid surgery.

## Discussion

From our systematic review and meta-analyses, a TV repair at the time of MV surgery in patients with moderate or less-than-moderate TR did not impact perioperative or postoperative all-cause mortality. Moreover, we observed a significant reduction in TR severity and TR progression following an intervention. However, no significant improvement in the NYHA functional classification was observed.

Previous research reported that increasing TR grades were associated with an increased risk of death in non-surgical populations, regardless of pulmonary hypertension or ejection fraction [11], whereas isolated moderate or severe TR was associated with an increased risk of death, regardless of cardiovascular or comorbid conditions [12]. Additionally, untreated TR increased mortality risks in patients undergoing left-sided transcatheter valve procedures, with two-fold mortality risk increases in patients with significant untreated TR and severe aortic stenosis undergoing transcatheter aortic valve replacement [13], and moderate to severe TR, which independently predicted death and re-hospitalisation at 12 months in percutaneous mitral intervention patients [14]. Although it is unclear how TR decreases survival, it is most likely related to impaired RV function. Along with survival, it has been demonstrated that TR may have a detrimental effect on functional status, as untreated moderate or greater TR was a risk factor for worse midterm survival and a higher NYHA class in a propensity-matched analysis when compared with De Vega tricuspid repair [15]. Although the same effects of TR progression may be inferred in our study population, a short-term follow-up of approximately two years did not provide enough information on long-term mortality and morbidity effects of TR progression.

The analysis of the studies included did not allow for a comparison between different etiologies of TR, as only two of the studies compared TR in these different settings with one study including only degenerative disease. Previous studies have reported contrasting results in TR progression after MVR in some subgroups [16], particularly regarding degenerative disease with some studies failing to show a correlation between tricuspid annular dimensions and TR progression [17]. As such, care must be taken, in future research, to differentiate between these different pathological settings and avoid generalization in treatment indications.

Additional valvular procedures carry increased risk and operative mortality [18]. Nevertheless, our meta-analysis did not identify added morbidity or mortality in patients undergoing MV surgery and concomitant TV repair. This finding was supported by a Society of Thoracic Surgeons Adult Cardiac Surgery Database analysis, which showed no increase in risk-adjusted operative mortality for TV repair at all TR grades, suggesting concomitant tricuspid surgery may not confer added mortality risk [10]. Conversely, operative risks for patients requiring a subsequent reoperation for residual/recurrent TR remained as high as 35% [19]. Gammie et al*.* has planned to conduct a 5-year follow-up, which may delineate long-term clinical effects.

Dreyfus et al*.* concluded that remodelling TV annuloplasty based on tricuspid dilation improved functional status irrespective of the regurgitation grade. The authors intraoperatively measured the tricuspid annular diameter from the anteroseptal to the antero-posterior commissure. Patients with a tricuspid annular dimension ≥ 70 mm undergoing tricuspid annuloplasty had an improved functional status. However, a tricuspid annulus diameter ≥ 40 mm or > 21 mm/m^2^ was proposed as an alternative cut-off as this value accurately identified patients undergoing MV repair without concomitant TV annuloplasty and who had poor echocardiographic outcomes, including significant TR and a lack of RV reverse remodelling at follow-up [20].

In terms of adverse events, Gammie et al*.* reported a substantially higher rate of permanent pacemaker implantation [21]. TV annuloplasty was previously associated with higher rates of this pacemaker necessity [22], which were associated subsequently with device malfunction, thrombosis, infection, recurrent or progressive TR, RV remodelling and reduced survival risks [23]. However, no other selected study reported pacemaker implantation rates.

## Study limitations

Some limitations were identified in our investigation. Firstly, only four studies were selected for analysis, however, these represent the totality of RCTs currently published on the subject, and the potential bias risk was considered accordingly. Secondly, we did not have access to individual patient data. Thirdly, heterogeneous TR-group inclusion criteria and several TV repair techniques were used in different studies; however, significant heterogeneity was not identified in our analyses. Fourthly, several studies did not report adverse events, especially pacemaker implantation rates, which should be factored into shared decision-making with patients. Lastly, the average follow-up period was 1–3.8 years, thus, we suggest that significant differences in functional status and mortality may be observed over a longer time-frame due to TR progression and consequent RV remodelling\failure.

## Conclusions

Our pooled analyses suggested that a tricuspid-valve repair at the time of MV surgery in patients with moderate or less-than-moderate TR did not impact perioperative or all-cause mortality, despite reducing TR severity and TR progression following intervention. To the authors knowledge this is the first systematic review and meta-analysis performed on this subject.

## Supplementary Information

**Additional file 1:** Funnel Plots and Supplemental Tables.

## Data Availability

All data generated or analyzed during this study is included in this published article [and its Additional file 1].

## Abbreviations

ITT: Intention-to-treat
NYHA: New York Association
OR: Odds Ratio
PRISMA: Preferred Reporting Items for Systematic Reviews and Meta-Analysis
RCTs: Randomised Clinical Trials
TR: Tricuspid Regurgitation
TV: Tricuspid Valve
